# Myxoid pleomorphic liposarcoma of the falciform ligament: a rare case report

**DOI:** 10.1093/jscr/rjac531

**Published:** 2022-12-07

**Authors:** Bashayer AlObaid, Nayef A Alzahrani, Nada Shokor, Kanan Alshammari

**Affiliations:** College of Medicine, King Saud bin Abdulaziz University for Health Sciences, Riyadh, Saudi Arabia; Department of Surgery, National Guard Health Affairs, King Abdulaziz Medical City, Riyadh, Saudi Arabia; Department of Surgery, St George Hospital & University of New South Wales, Sydney, New South Wales, Australia; King Abdulaziz Medical Center, Department of Pathology and Laboratory Medicine, Riyadh, Saudi Arabia; King Abdulaziz Medical Center, Department of Oncology, Riyadh, Saudi Arabia

## Abstract

Liposarcomas are a group of malignancies that mainly affect adults. Myxoid pleomorphic liposarcoma (MPL) is a newly added subtype of liposarcomas [1]. It is extremely rare and mostly affects infants and children, and it has a predilection for the mediastinum. We report a case of a 58-years-old female with MPL originating from the falciform ligament who presented initially with vague abdominal pain. MPLs have an aggressive pathology and high metastasis and recurrence potential.

## INTRODUCTION

Myxoid pleomorphic liposarcoma (MPL) is an extremely rare type of liposarcomas, which has a highly aggressive malignant pathology [1]. It is characterized by a combination of histological features of both pleomorphic and myxoid liposarcomas. The neoplasm almost exclusively occurs in children, and it has a predilection for mediastinum. We describe a case that is, to our knowledge, the first to report MPL originating from the falciform ligament of an adult patient.

## CASE PRESENTATION

A 58-year-old female presented to the outpatient clinic with a 1-year history of vague abdominal pain associated with nausea and vomiting. She denied any changes in her bowel habits. Her past medical history includes Type 2 diabetes mellitus, dyslipidemia and hypertension. On examination, she had mild generalized abdominal tenderness without guarding or rebound tenderness. No enlargement in the lymph nodes. On laboratory analysis, a full blood work-up was done including complete blood count, electrolytes, liver function test and they were normal. The tumor markers CEA, CA19-9 and CA 125 were also normal.

**Figure 1:**
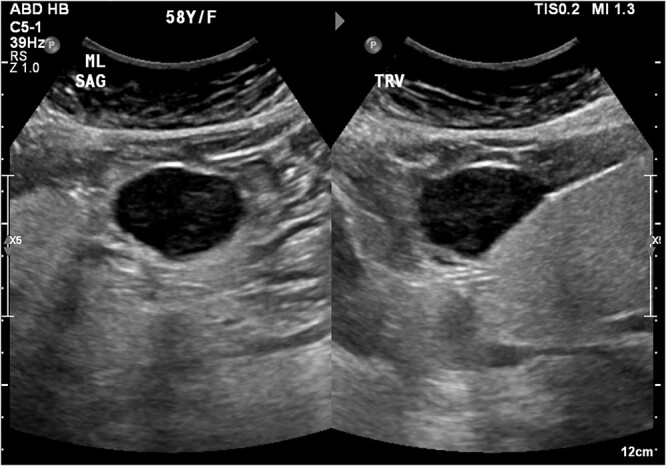
Homogonous hypoechoic lesion anterior to the left hepatic lobe. Color doppler images (not shown) shows internal blood flow.

**Figure 2:**
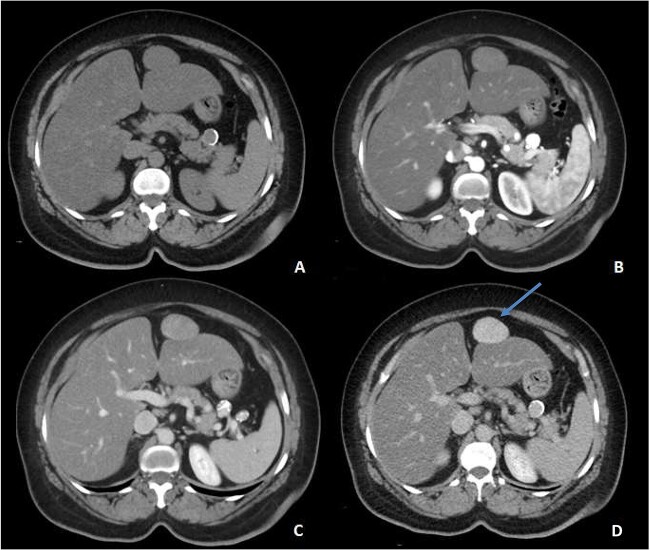
Pre-contrast (**A**), Arterial (**B**), Porto-venous (**C**) and Delayed (**D**) images of contrast enhanced CT demonstrating a well-defined, homogeneous, progressively enhancing mass anterior to the left hepatic lobe, with homogeneous delayed images (blue arrow). Incidental hepatic steatosis and a small splenic artery aneurysm are present.

An ultrasound of the abdomen revealed a homogonous hypoechoic lesion anterior to the left hepatic lobe (Fig. 1). Color doppler images (not shown) shows internal blood flow. A subsequent abdominal computed tomography (CT) scan was done, and it revealed a 4.9 × 5.5 cm well-defined, homogeneous, progressively enhancing mass anterior to the left hepatic lobe (Fig. 2). The differential diagnoses included desmoid tumor, leiomyoma, hemangioma and gastrointestinal stromal tumor (GIST). EGD and CT colonography were done and both came back negative for synchronous lesion.

**Figure 3:**
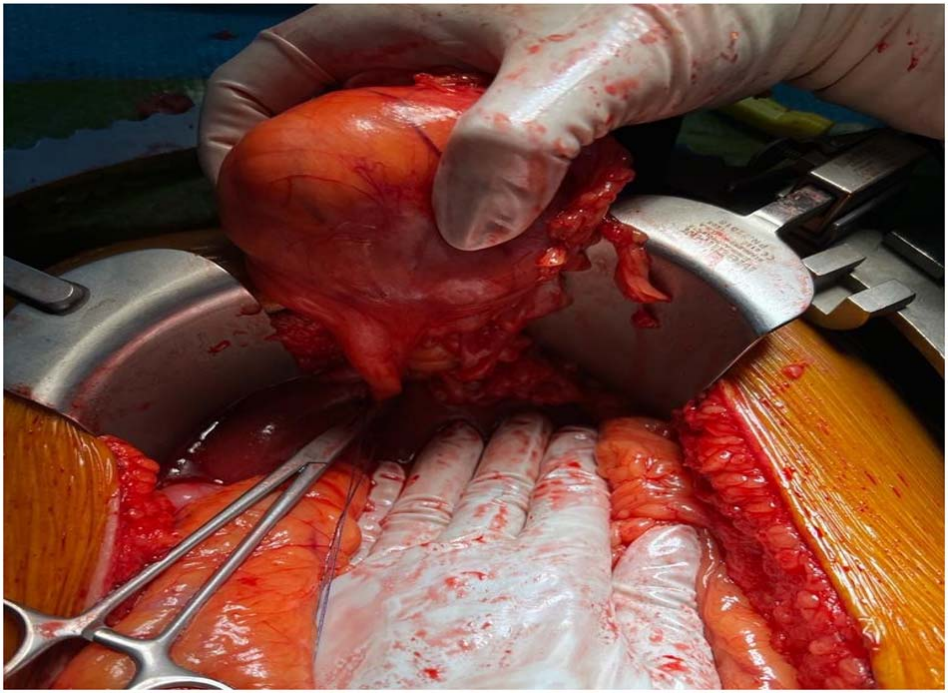
Macroscopic view of the tumor prior to resection.

The patient underwent exploratory laparotomy. The surgical exploration revealed a mass originating from the falciform ligament measuring around 6 × 6 cm that was soft in nature (Fig. 3). There was no peritoneal metastasis. The tumor was completely resected, and the specimen was sent for histopathology evaluation. The post-operative recovery was uneventful.

**Figure 4:**
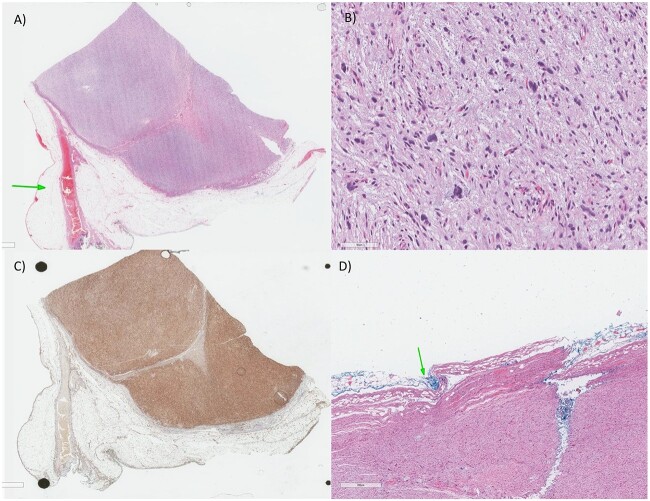
(**A**) Low magnification: there is a highly cellular process despite being well-circumscribed from the surrounding adipose tissue and vascular structures (arrow). (**B**) Higher magnification: The tumor is composed of highly atypical cells, some with bizarre dark nuclei, in a bluish hue (myxoid stroma). (**C**) The tumor has only a strong tumor positivity for the CD34, CD99 and Vimentin (mesenchymal markers), indicative of a sarcoma process (CD34 in this image). (**D**) For the histopathology report, as there’s a debate for chemotherapy role in soft tissue sarcoma, the size (larger than 5 cm—our case is 6.0 cm), grade (Grade 3—our case is high-grade), margin (arrow) and location as considered determinants for adjuvant chemotherapy.

Histologically, on initial evaluation, the resected mass was measured 7.0 × 6.5 × 5.0 cm and it was composed of fibrofatty tissue and peritonized surface. Microscopic examination demonstrated that the tumor is composed of cellular sheets of highly atypical pleomorphic cells. (Fig. 4B) The tumor cells are hyperchromatic, some with bizarre nuclei, in a background of myxoid stroma, all consistent with high-grade sarcoma. A panel of immunohistochemical stains was done to further differentiate the tumor. The tumor cells were negative for CD117, DOG-1, SMA, Myogenin, Calretinin, WT-1, HMB45, SOX-10 and S100, which exclude GIST (gastrointestinal stromal tumor), smooth and skeletal muscle, mesothelial, melanocytic/malignant PECOMA and neural differentiation, respectively. The tumor cells were positive for CD99, Vimentin and CD34 (Fig. 4C), which is consistent with a mesenchymal differentiation and sarcoma. In immunohistochemistry, there was loss of nuclear staining for RB1. In addition, there was lack of MDM2 amplification by FISH and lack of sarcoma-specific fusions by Sarcoma Targeted Gene Fusion Panel (SARCP) testing. Therefore, the diagnosis of the omental mass is MPL.

## DISCUSSION

Liposarcoma is a type of malignancy that affects the fat tissue. According to World Health Organization (WHO), liposarcomas were initially classified into well-differentiated, dedifferentiated, pleomorphic and myxoid [1]. However, new entities were added to the classification. MPL is a recently added subtype of liposarcomas that was recognized in 2009 by Alaggio *et al*. [2]. It is defined as a malignancy showing mixed histological characteristics of a conventional myxoid liposarcoma and a pleomorphic liposarcoma [3]. It is extremely rare and mostly affects infants and children, and it has a predilection for the mediastinum but can happen in other sites. However, it is extremely rare to originate from the falciform ligament. Because of the rarity of MPLs, they have not been studied extensively. There is no recognized cytogenic or molecular genetic abnormality associated with the disease has been found [4]. However, the current data available shows that MPLs lack the translocations linked with conventional myxoid liposarcoma. MPLs also lack MDM2 amplification and shows inactivation of RB1 tumor suppressor gene. The neoplasm has an aggressive nature, a high risk of metastasis and recurrence, and a low survival rate.

## CONFLICT OF INTEREST STATEMENT

None declared.

## FUNDING

None.
